# QSAR study and rustic ligand-based virtual screening in a search for aminooxadiazole derivatives as PIM1 inhibitors

**DOI:** 10.1186/s13065-018-0401-x

**Published:** 2018-03-22

**Authors:** Adnane Aouidate, Adib Ghaleb, Mounir Ghamali, Samir Chtita, Abdellah Ousaa, M’barek Choukrad, Abdelouahid Sbai, Mohammed Bouachrine, Tahar Lakhlifi

**Affiliations:** 1 MCNSL, School of Sciences, Moulay Ismail University, Meknes, Morocco; 2 High School of Technology, Moulay Ismail University, Meknes, Morocco

**Keywords:** PIM1, Aminooxadiazoles, QSAR model, Applicability domain, MLR, Virtual screening

## Abstract

**Background:**

Quantitative structure–activity relationship (QSAR) was carried out to study a series of aminooxadiazoles as PIM1 inhibitors having p*k*_i_ ranging from 5.59 to 9.62 (*k*_*i*_ in nM). The present study was performed using Genetic Algorithm method of variable selection (GFA), multiple linear regression analysis (MLR) and non-linear multiple regression analysis (MNLR) to build unambiguous QSAR models of 34 substituted aminooxadiazoles toward PIM1 inhibitory activity based on topological descriptors.

**Results:**

Results showed that the MLR and MNLR predict activity in a satisfactory manner. We concluded that both models provide a high agreement between the predicted and observed values of PIM1 inhibitory activity. Also, they exhibit good stability towards data variations for the validation methods. Furthermore, based on the similarity principle we performed a database screening to identify putative PIM1 candidates inhibitors, and predict their inhibitory activities using the proposed MLR model.

**Conclusions:**

This approach can be easily handled by chemists, to distinguish, which ones among the future designed aminooxadiazoles structures could be lead-like and those that couldn’t be, thus, they can be eliminated in the early stages of drug discovery process.
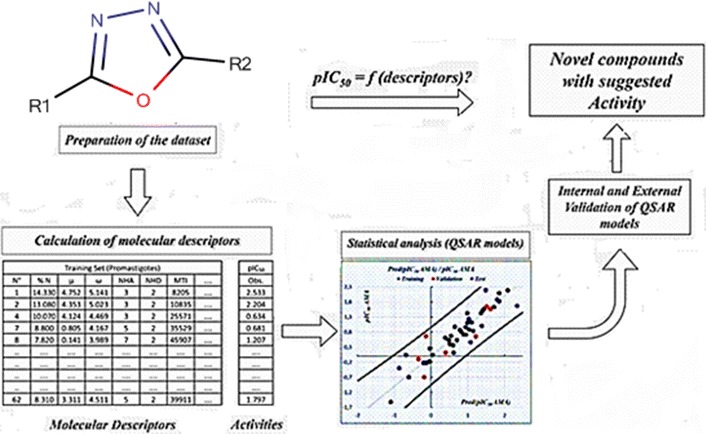

## Introduction

Proviral integration site for Moloney murine leukemia virus (PIM) is a family of serine/threonine protein kinases that are widely expressed and are involved in cell survival and proliferation as well as a number of other signal transduction [1, 2]. This family is composed of three isoforms: PIM1, PIM2, and PIM3 that share a high level of sequence homology and exhibit some functional redundancy. Over-expression of PIM1 and PIM2 kinases has been reported in hematologic malignancies also in solid tumors such as diffuse large B cell lymphomas (DLBCL) and prostate cancer [3], thus, these findings make it an attractive target for cancer therapy [1].

Several heterocycles have been studied with different approaches so far, as 5-(1H-indol-5-yl)-1,3,4-thiadiazol-2-amines [4] and pyrrolo carbazole [5], thiazolidine [6] including many clinical compounds as SGI-1776 [7] and AZD-1208 [8] that have been found to be able to inhibit PIM1 kinase and exhibit an anti-cancer activity. However, no PIM1 inhibitor has crossed all stages of drug discovery process and approved as a drug yet, therefore there is always a need to discover and identify new PIM1 inhibitors. Consequently, in order to reduce time and cost, in addition to design and identify more potent PIM inhibitors, theoretical research can circumvent these difficulties and allow obtaining precise data while taking advantage of the rapid progress of computing chemical descriptors, which can be obtained easily from publicly available software and servers. Descriptors can be exploited to build a quantitative structure–activity relationship (QSAR) model to enable calculation of the activity and prediction of the efficacy of new potent aminooxadiazoles. In the recent years, many QSAR studies have been developed on different PIM1 heterocycle inhibitors [9, 10], despite, it would be worthwhile to extend these data and develop QSAR studies on new PIM1 inhibitors. Recently, a series of some potent PIM1 inhibitors: have been designed and reported by Wurz et al. [11]. We believe that this is the first QSAR study performed on the reported activities of this series. That prompted us to aim an in silico study based on it to design new molecules with enhanced inhibitory activity.

Quantitative structure activity relationship is one of the most common approach in computer aided drug design [12] as well as in many other applications, including predictive toxicology, and risk assessment [13, 14]. QSAR studies are based on the fact that the biological activities of organic molecules depend on their chemical structures, and can be quantitatively described by chemometrics models. This approach has a wide application for evaluating the potential impact of chemicals on human health, and technological processes as in the pharmaceutical industry and drug discovery [15]. Thus, it is necessary to develop a QSAR model for the prediction of activity before synthesis of new PIM1 inhibitors. A successful QSAR model not only, helps to understand relationships between the structural properties and biological activity of any class of molecules, but also provides researchers a deep analysis about the lead molecules to be used in further studies [16].

The present study aims to derive QSAR models, which explain the relationship between the anti-cancer activity and the structure of 34 compounds based on physicochemical descriptors using several chemometric methods such as genetic functional algorithm for variable selection GFA, multiple linear regression MLR and non-linear regression MNLR for modeling and William’s plot for applicability domain. Finally, PubChem database was virtually screened using the most active compound in the series as a reference molecule.

## Materials and methods

**Fig. 1:**
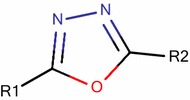
The chemical structure of the studied compounds

For QSAR studies a series of 34 aminooxadiazoles with reported activity values were compiled from the literature [11]. The activity was expressed as *k*_i_ and is defined as the binding affinity constants of aminooxadiazoles to PIM1 kinase. Because the inhibitory activity values cover a wide range, they are converted into logarithm units (p*k*_i_= − log *k*_*i*_) (*k*_i_ in nM) for modelling purposes. Figure 1 and Table 1 show the substituted structures of the studied compounds. For modeling, the data set was split into two sets. Twenty-seven molecules were chosen based on the activity variation to represent the quantitative model (training set) and the rest were used to test the performance of proposed model (Test set). Additionally leave-one-out protocol and Y-Randomization were performed on the training set for internal validation of the obtained models.

**Table 1:**
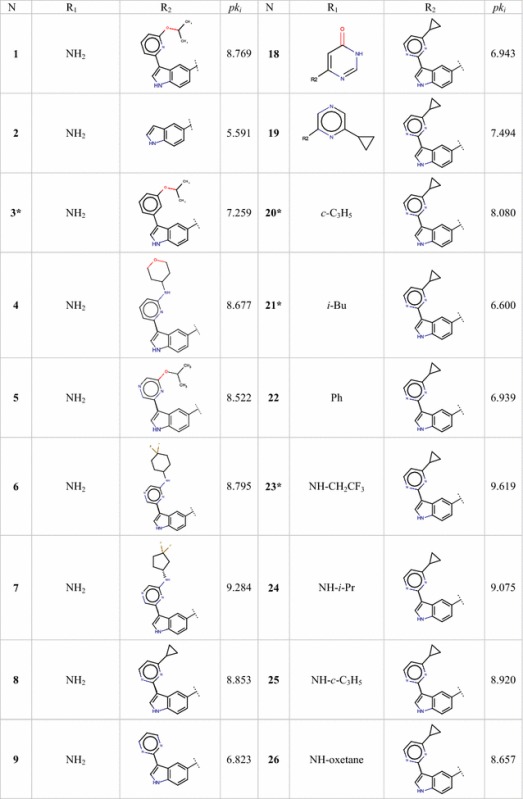

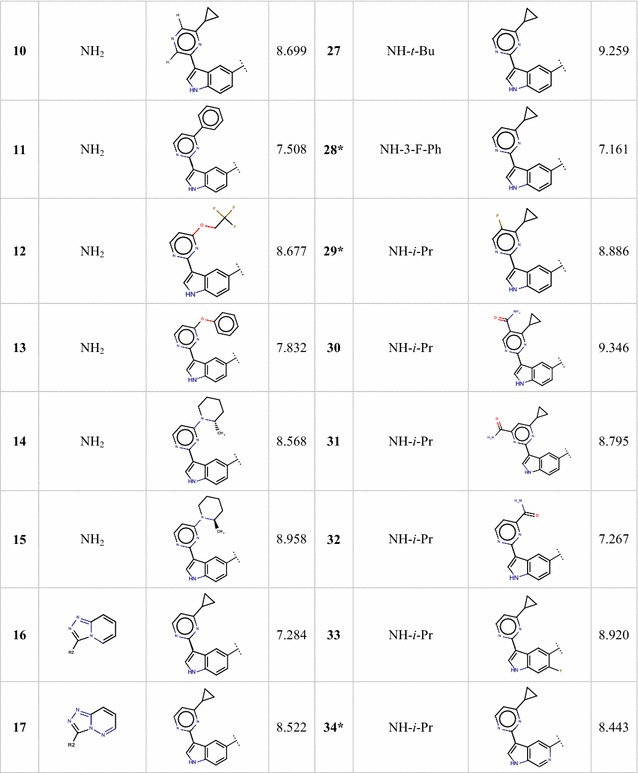
Observed activities of studied aminooxadiazoles

*Test set

### Molecular modeling

All modeling studies were performed using the SYBYL-X 2.0 molecular modeling package (Tripos Inc., St. Louis, USA) running on a windows 7, 32 bit workstation. Three-dimensional structures were built using the SKETCH option in SYBYL. All compounds were minimized under the Tripos standard force field [17] with Gasteiger–Hückel atomic partial charges [18] by the Powell method with a gradient convergence criterion of 0.01 kcal/mol Å. To describe the compound structural diversity and in order to obtain validated QSAR models. The optimized structures were saved in sdf format, and transferred to PaDEL-Descriptor version 2.18 tool kits, topological descriptors encode the chemical properties have been calculated for each aminooxadiazole, using PaDEL server [19]. Only three suitable ones have been chosen as relevant descriptors for the studied inhibitory activity: Mannhold LogP (MLogP) and two Burden modified eigenvalues (SpMax1_Bhi and SpMin6_Bhm).

### Methodology

After the calculation of descriptors, a Genetic Function Algorithm (GFA) analysis was performed to select the relevant molecular descriptors [20, 21]. The selected descriptors were then used to perform an MLR study until a valid model including: the critical probability P value < 0.05 for all descriptors and for the complete model, The Fisher static, the coefficient of determination, the mean squared error and the multi-collinearity test, internal and external validations, in addition to the Y-Randomization. Those selected descriptors were exploited to generate the applicability domain, then to evaluate a non-linear model. Later, the proposed model was used to identify aminooxadiazoles analogues in PubChem database and predict their PIM1 inhibitory activities.

### Statistical analysis

In the present study, XLSTAT version 2013 [22] was used to perform multiple linear regression (MLR) and non-linear regression (MNLR), which are two statistical methods used to derive a mathematical relationship between a property of a given system and a set of descriptors that encode chemical information. A Genetic Algorithm tool was used to carried out the Genetic algorithm analysis (GFA) to reduce the number of the variables of the data set and choose the pertinent ones, in which, the mutation probability and smoothing parameter were set to 0.1 and 0.5, respectively. GFA in this study serves to select descriptors that were applied as input in multiple linear regression (MLR), multiple non-linear regression (MNLR) and applicability domain (AD).

### Validation

The main objective of a QSAR study is to obtain a model with the highest predictive and generalization abilities. In order to evaluate the predictive ability of the developed QSAR models, two principals (internal validation and external validation) were performed. For the internal validation the leave-one-out cross-validation (Q^2^) was used to evaluate the internal stability and of the present models. A high Q^2^ value means a high internal predictive power of a QSAR model and a good robustness. Nevertheless, the study of Globarikh [23] indicated that there is no correlation between the value of Q^2^ for the training set and predictive ability of the test set, revealing that the Q^2^ is still insufficient for a reliable estimation of the model’s predictive power for all new compounds. Thus, the external validation remains the only way to determine both the generalizability and the true predictive ability of QSAR models for new chemicals. For this reason, the statistical external validation was applied to the models as described by Globarikh and Tropsha. Roy and Roy [23–25] using a test set.

### Y-Randomization test

The obtained models were further validated by the Y-Randomization method [21]. The dependent vector (p*k*_i_) is randomly shuffled many times and after every iteration, a new QSAR model is developed. The new QSAR models are expected to have lower Q^2^ and R^2^ values than those the original models. This technique is carried out to eliminate the possibility of the chance correlation. If higher values of the Q^2^ and R^2^ are obtained, it means that an acceptable QSAR can’t be generated for this data set because of the structural redundancy and chance correlation.

## Results and discussion

### Data set for analysis

A QSAR study was carried out on 34 aminooxadiazoles for the first time in order to establish a quantitative relationship between the PIM1 inhibitory activity and their chemical structures. The three selected descriptors by GFA method among 1543 other ones firstly calculated by PaDEL server are shown in Table 2.

**Table 2:** The values of three relevant molecular descriptors used in the best QSAR model

No	*pk*_*i*_	SpMin6_Bhm	MLogP	SpMax1_Bhi	No	*pk*_*i*_	SpMin6_Bhm	MLogP	SpMax1_Bhi
**1**	8.769	1.276	2.67	4.187	**18**	8.522	1.352	2.890	4.225
**2**	5.591	0.707	2.01	4.135	**19**	6.943	1.228	2.889	4.172
3*	7.259	1.276	2.889	4.188	20*	7.494	1.372	3.330	4.190
**4**	8.677	1.361	2.78	4.188	21*	8.080	1.372	3	4.191
**5**	8.522	1.266	2.45	4.187	**22**	6.600	1.364	3.11	4.190
**6**	8.795	1.361	2.56	4.188	23*	6.939	1.350	3.329	4.194
**7**	9.284	1.349	2.449	4.188	**24**	9.619	1.334	2.449	4.190
**8**	8.853	1.286	2.56	4.189	**25**	9.075	1.340	2.89	4.190
**9**	6.823	1.015	2.23	4.186	**26**	8.920	1.372	2.89	4.190
**10**	8.699	1.295	2.56	4.190	**27**	8.657	1.341	2.78	4.190
**11**	7.508	1.015	2.01	4.188	**28**	9.259	1.341	3	4.190
**12**	8.677	1.278	2.89	4.194	29*	7.161	1.350	3.11	4.190
**13**	7.832	1.276	2.78	4.187	30*	8.886	1.338	2.78	4.191
**14**	8.568	1.361	2.78	4.188	**31**	9.346	1.349	2.78	4.192
**15**	8.958	1.361	2.78	4.188	**32**	8.795	1.341	2.78	4.192
**16**	7.284	1.360	3.11	4.226	**33**	7.267	1.249	2.45	4.188
**17**	8.522	1.276	2.67	4.187	34*	8.920	1.335	2.78	4.195

* Test set

### Multiple linear regression (MLR)

Based on the selected descriptors a mathematical linear model was proposed to predict quantitatively the physicochemical effects of substituents on the PIM1 inhibitory activity of the 34 molecules using multiple linear regression. The linear model using this method includes three molecular descriptors: the total energy SpMin6_Bhm, the energy MLogP and the surface tension SpMax1_Bhi.

$$Y = a_{0} + \mathop \sum \limits_{i = 1}^{n} a_{i} x_{i}$$

The following equation represents the best obtained linear QSAR model using the regression linear multiple (MLR) method:

1 $$pK_{i} = 43.24 + 8.396 \times \left( { \varvec{SpMin}6\_\varvec{Bhm}} \right) - 1.93 \times \left( {\varvec{MLogP}} \right)\varvec{ } - 9.65 \times \left( {\varvec{SpMax}1\_\varvec{Bhi}} \right)\varvec{ }$$

N = 27, R = 0.838, R^2^ = 0.714, Q^2^ = 0.60, MSE = 0.29, F = 19.12, P < 0.0001.

The established models are judged by the statistical keys, such as, R^2^ is the coefficient of determination, F is the Fisher statistic and MSE is the mean squared error. Higher coefficient of determination and lower mean squared error indicate that the model is more reliable. A P smaller than 0.05 means that the obtained equation is statistically significant at the 95% level. The leave one out cross-validated correlation coefficient LOO (Q^2^ = 0.60) illustrates the reliability of the model by focusing on the sensitivity of the model towards the elimination of any single data point. A value of Q^2^ greater than 0.5 is the basic criteria to qualify a model as valid [23].

The multi-collinearity between the three chosen descriptors was evaluated by calculating their variation inflation factors VIF as shown in Table 3. The VIF [26] was defined as 1/(1 − R^2^), where R is the coefficient of correlation between one descriptor and all the other descriptors in the proposed model. A VIF value greater than 5.0 indicates that the model is unstable; a value between 1.0 and 4.0 indicates that the model is acceptable. Accordingly, it has been found that the descriptors used in the proposed model have very low-inter-correlation.

**Table 3:** Multi-colinearity test

Variables	SpMin6_Bhm	MLogP	SpMax1_Bhi
VIF	3.035	2.201	1.869

Negative values in the regression coefficients show that the indicated variables (MLogP and SpMax1_Bhi) contribute negatively to the value of p*k*_i_, whereas positive value in the regression coefficient of variable (SpMin6_Bhm) indicates that the greater the value of the variable, the greater the value of the p*k*_i_.

**Fig. 2:**
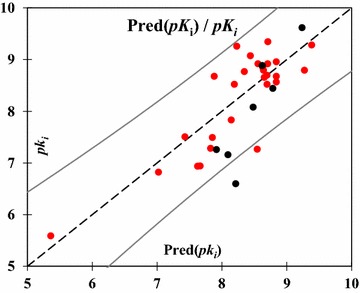
Graphical representation of predicted and observed activity (p*k*_i_) values calculated by MLR

The predicted values computed using this MLR model with the experimental values for the training and test sets are shown in Table 4, and plotted in Fig. 2. The selected descriptors (Eq. 1) in the MLR model are then used as the input variables to perform the multiple nonlinear regression (MNLR).

**Table 4:** Observed values and calculated values of p*k*_i_ according to different methods

No	*pk*_*i*_ (obs)	*pk*_*i*_ (pred)	
		MLR	MNLR
**1**	8.769	8.345	8.328
**2**	5.591	5.358	5.535
3*	7.259	7.916	8.045
**4**	8.677	8.838	9.037
**5**	8.522	8.695	8.261
**6**	8.795	9.272	9.197
**7**	9.284	9.383	9.036
**8**	8.853	8.629	8.514
**9**	6.823	7.021	7.510
**10**	8.699	8.694	8.595
**11**	7.508	7.429	6.996
**12**	8.677	7.880	8.171
**13**	7.832	8.143	8.186
**14**	8.568	8.838	9.024
**15**	8.958	8.838	9.024
**16**	7.284	7.824	7.648
**17**	8.522	8.192	8.241
**18**	6.943	7.664	7.010
**19**	7.494	7.852	7.486
20*	8.080	8.481	8.772
21*	6.600	8.211	8.299
**22**	6.939	7.623	7.309
23*	9.619	9.235	8.919
**24**	9.075	8.439	8.655
**25**	8.920	8.706	9.016
**26**	8.657	8.653	8.856
**27**	9.259	8.228	8.390
28*	7.161	8.092	8.144
29*	8.886	8.621	8.856
**30**	9.346	8.708	8.984
**31**	8.795	8.641	8.909
**32**	7.267	8.544	8.174
**33**	8.920	8.556	8.902
34*	8.522	8.785	8.804

* Test set

### Multiples non-linear regression (MNLR)

The nonlinear regression model was also used to evaluate the effect of the substituents in the studied aminooxadiazoles on the PIM1 inhibitory activity, improve the structure–activity relationship in quantitative manner.

Training set used in MLR and descriptors selected by GFA were used in this method to build the non-linear model. The best regression performance was selected according to the coefficient of determination R^2^ and the mean squared error MSE, a pre-programmed function in the XLSTAT was used to evaluate the nonlinear regression model as follows:

$$y = = a + \left( {b X_{1} + c X_{2} + d X_{3} + e X_{4} \ldots } \right) + \left( {f X_{1}^{2} + gX_{2}^{2} + h X_{3}^{2} + i X_{4}^{2} \ldots } \right).$$

where *X*_*1*_*, X*_*2*_*, X*_*3*_*, X*_*4*_*…:* represent the variables, and *a, b, c, d…:* represent the parameters.

The resulting equation is as follows:

2 $$\begin{aligned} pK_{i} =& - 19641.39 - 47.63 \times \left( {\varvec{SpMin}6\_\varvec{Bhm}} \right) + 15.79 \times \left( {\varvec{MLogP}} \right) \hfill \\ & + 9356.96 \times \left( {\varvec{SpMax}1\_\varvec{Bhi}} \right) + \varvec{ }21.6\varvec{ } \times \varvec{ }\left( {\varvec{SpMin}6\_\varvec{Bhm}} \right)^{2} \hfill \\& - \varvec{ }3.10 \times \varvec{ }\left( {\varvec{MLogP}} \right)^{2} - 1113.59 \times \left( {\varvec{SpMax}1\_\varvec{Bhi}} \right)^{2} \hfill \\ \end{aligned}$$

N = 27, R = 0.910, R^2^ = 0.812, Q^2^ = 0.56, MSE = 0.22.

The leave one out cross-validated correlation coefficient LOO (Q^2^ = 0.56) illustrates the reliability of the model by focusing on the sensitivity of the model towards the elimination of any single data point. A value of Q^2^ greater than 0.5 is the basic criteria to qualify a model as valid [23]. It can be seen clearly from the key statistical indicators, coefficient of determination R^2^, mean squared error MSE and, value of Q^2^, that the predicting ability of this model is better than that of the linear model (MLR). The enhancement in the predictive ability was due to the involvement of the squared terms in the nonlinear model.

**Fig. 3:**
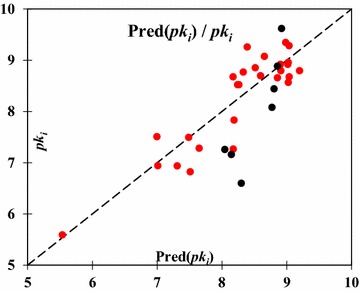
Graphical representation of predicted and observed activity (*pk*_*i*_) values calculated by MNLR

The predicted values computed using this MNLR model for the training and test sets are shown in Table 4, and plotted in Fig. 3.

### Applicability domain

The utility of a QSAR model is its accurate prediction ability for new chemical, so, once the QSAR model is built, its domain of applicability (AD) must be defined. A model is considered valid only if it is able to make predictions within its training domain and only the prediction for new compounds falling within its applicability domain can be regarded credible and not model extrapolations. The most common method to define the AD, it is based on the determination of the leverage value of each compound [25]. The Williams plot [The plot of standardized residuals versus leverage values (*h*)] is used in the present study to visualize the AD of the QSAR model.

$$h_{i} = x_{i}^{T} \left( {X^{T} X} \right)^{ - 1} x_{i}$$

where the x_i_ is the descriptor vector of the considered compound, X is the descriptor matrix derived from the training set descriptor values, the threshold is defined as:

$$h^{*} = \frac{{3\left( {k + 1} \right)}}{n}$$

where n is the number of compound in the training set, k is the number of the descriptors in the proposed model, a leverage (*h*) greater than the threshold (h*) indicates that the predicted response is an extrapolation of the model and, consequently, it can be unreliable.

**Fig. 4:**
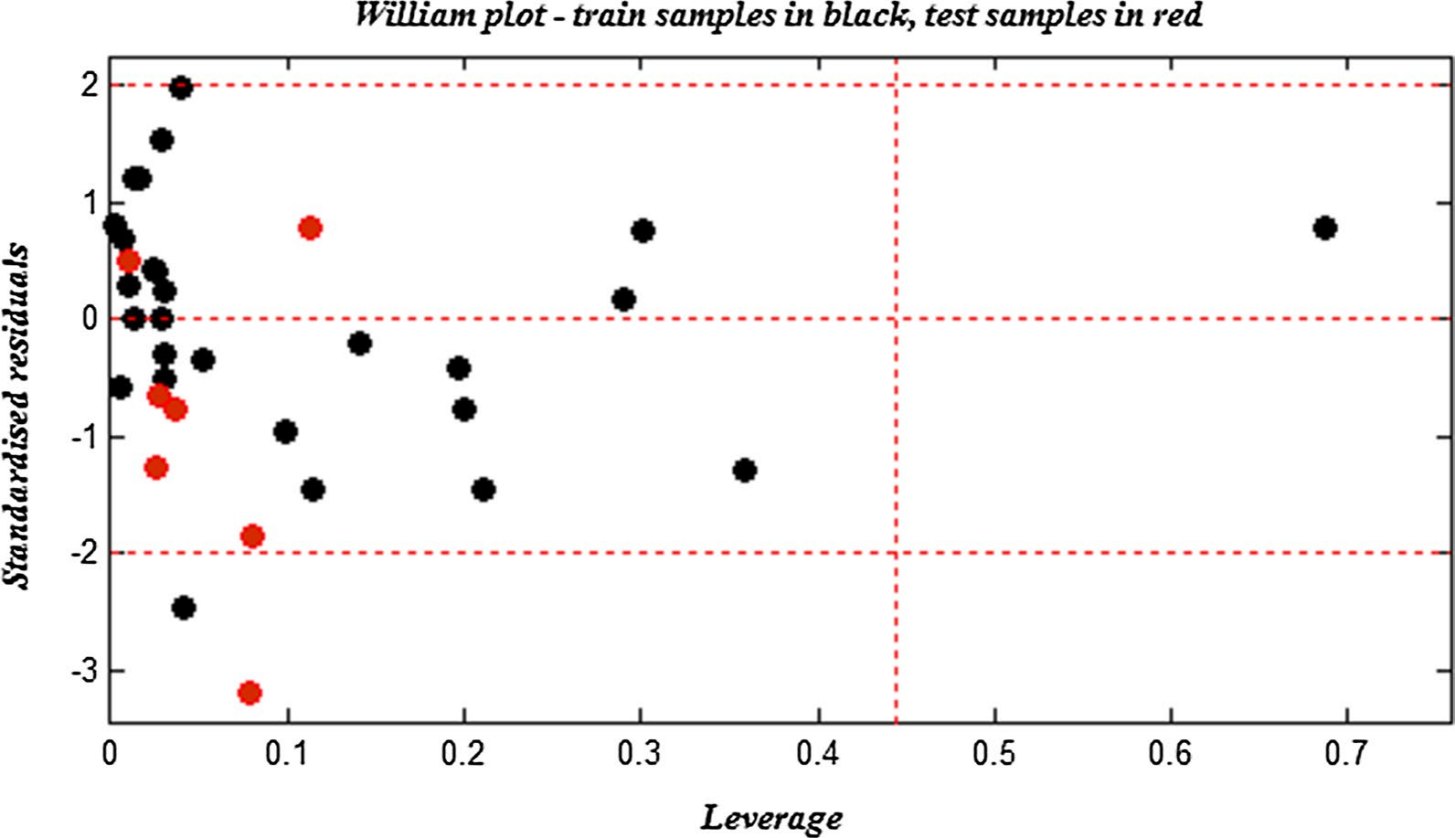
Williams plot for the training set and external validation for the PIM1 inhibitory activity of aminooxadiazole compounds, listed in Table 1 (h* = 0.44 and residual limits ± 2)

The Williams plot of the presented MLR model is shown in the Fig. 4, the applicability domain is established inside a squared area within ± 2 standard deviation and a leverage threshold h* of 0.44. As shown in the Williams plot the majority of the compounds in the data set are in this area, except one (Compound **2**) in training set exceeds the threshold and it is considered as an outlier compound. This erroneous prediction could probably be attributed to the R_2_ position, whereas, the majority of compounds are substituted by an indole linked to another moiety at this position this compound has just an indole moiety at the R_2_ position. Also, compound 22 in the test set is wrongly predicted (> 3 s), but with lower leverage values (*h* < h*) and that could probably be attributed to a different mechanism of action rather than to molecular structures [25].

### Y-Randomization

The Y-Randomization method was carried out to validate the MLR and MNLR models. Several random shuffles of the dependent variable (p*k*_*i*_) were performed then after every shuffle, a QSAR was developed and obtained results are shown in Table 5. The low Q^2^ and R^2^ values obtained after every shuffle indicate that the good result in our original MLR and MLR models are not due to a chance correlation of the training set.

**Table 5:** Q^2^ and R^2^ values after several Y-Randomization tests

Iteration	MLR		MNLR	
	Q^2^	R^2^	Q^2^	R^2^
1	0.390	0.235	0.031	0.350
2	0.120	0.094	0.095	0.008
3	0.290	0.124	0.079	0.190
4	0.340	0.129	− 0.264	0.290
5	0.180	0.263	− 0.160	0.335
6	0.160	0.194	− 0.522	0.140
7	0.20	0.075	− 0.105	0.006
8	0.130	0.043	0.230	0.026
9	0.140	0.116	0.120	0.196
10	0.230	0.031	0.060	0.131

### External validation

To test the prediction ability of the obtained models, it is required the use of a test set for external validation. Thus, the models generated on the training set using 26 aminooxadiazoles were used to predict the PIM1 inhibitory activity of the remaining molecules. The parameters of the performance of the generated models are shown in Table 6. It can be seen clearly that the MNLR is statically better than the MLR model in terms of coefficient of determination, but the MLR has a better predictive ability and good internal stability.

**Table 6:** The statistical results of MLR and MNLR models with validation techniques

Method/parameter	*R*	*R* ^*2*^	*Q* ^*2*^	$R_{test}^{2}$	MSE
MLR	0.838	0.712	0.60	0.81	0.29
MNLR	0.910	0.812	0.56	0.75	0.22

Among the obtained models for this series, the MLR model has the highest prediction ability for the test set ($R_{test}^{2}$ = 0.81), also the highest cross-validation coefficient (Q^2^ = 0.60), all that support the applicability of the proposed MLR prediction model. However, both the results obtained by the MLR and MNLR should be regarded as satisfactory for predicting the PIM1 activity using the proposed descriptors.

### Virtual screening for aminooxadiazole analogues and prediction of their PIM1 inhibitory activities

**Fig. 5:**
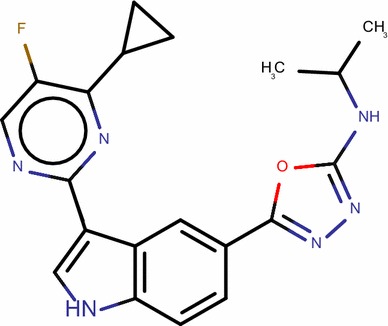
Reference structure of aminooxadiazole model with lowest binding constant *k*_i_

**Fig. 6:**
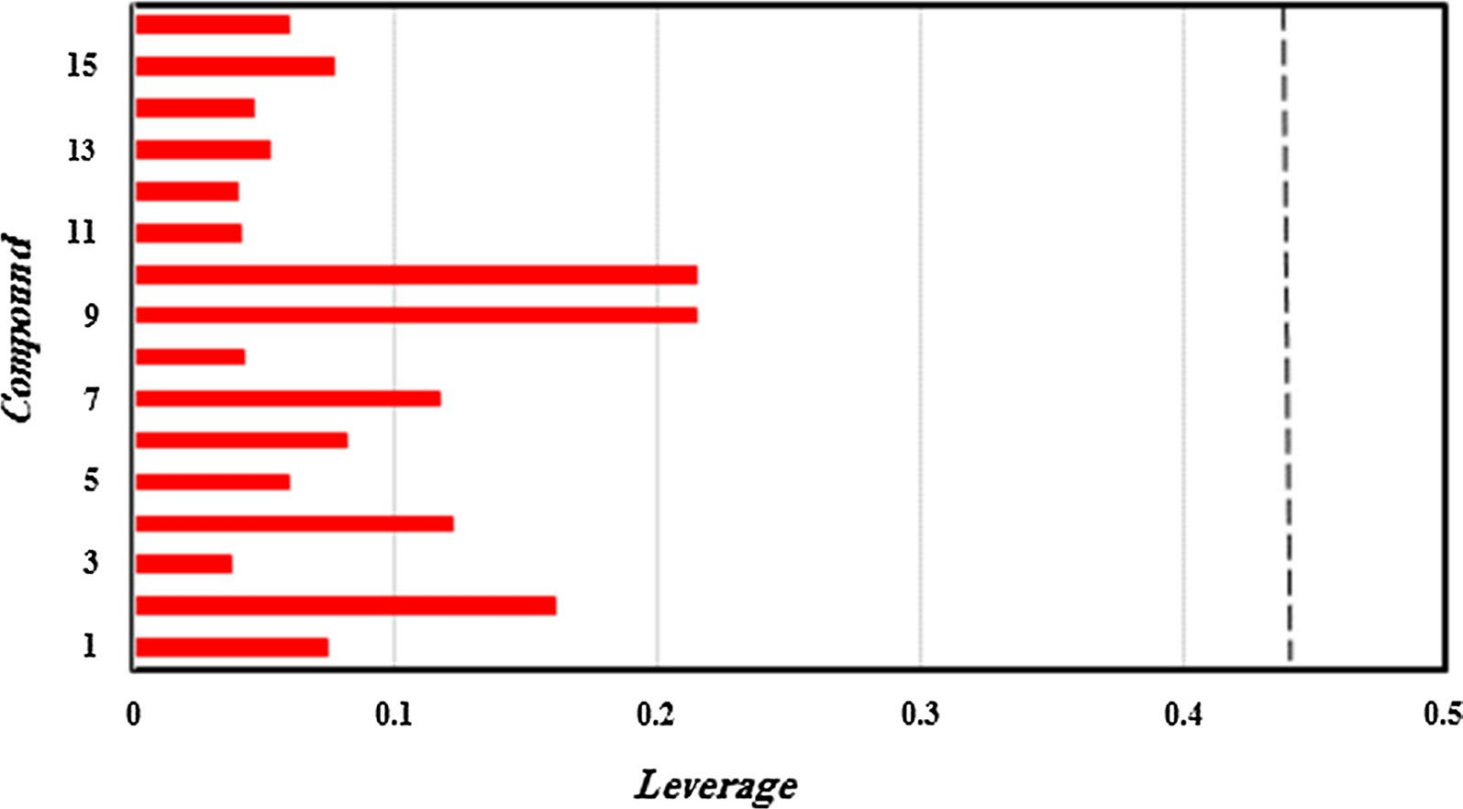
Leverage values of the screened compounds from the PubChem database for the PIM1 inhibitory activity, listed in Table 7 (h* = 0.44)

Overall, this study can be used to screen chemical databases to identify new PIM1 inhibitors as well as to predict their inhibitory activities. Therefore, the built MLR model was used to screen the PubChem database, by searching compounds had 95% similarity with the most active compound of the studied series (Compound **29**) and fulfilling the Lipinski’s rule of bioavailability [27]. Sixteen compounds were identified as shown in Table 7 and their p*k*_*i*_ values were predicted in addition to their leverages (*h*) to check if they fall in the AD of the proposed model. (Table 7, Figs. 5 and 6).

**Table 7:**
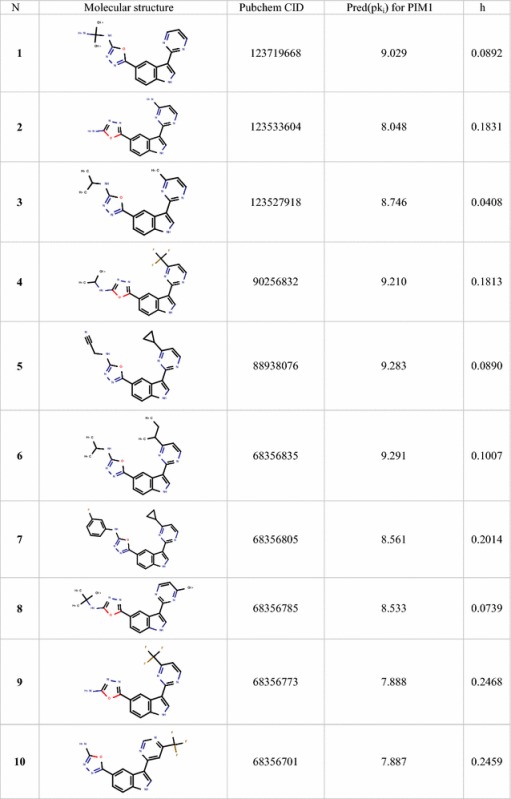

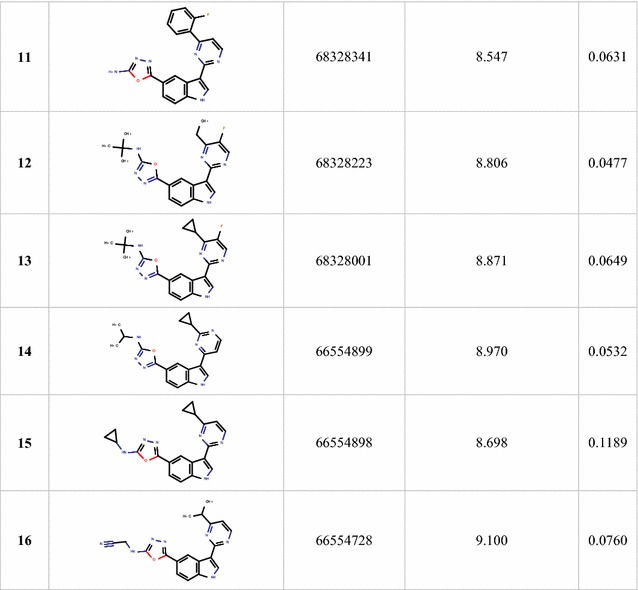
Predicted values and calculated *h* of *pk*_*i*_ (*k*_*i*_ in nM) of the sixteen identified hits

It can be seen from the Fig. 6 that all identified compounds have *h *< h*, (h* = 0.44) so their predicted values are regarded reliable.

## Conclusion

To predict the PIM1 inhibitory activity of a series substituted aminooxadiazoles, two unambiguous models were developed in this study with topological descriptors. A good stability and prediction ability were exhibited by MLR and MNLR models, on the same set of descriptor. Furthermore, the obtained results from each model on this series of compounds are quite similar, no one of the established models is considered better than the other. So, the MLR and MNLR models are regarded as effective tools to predict PIM1 inhibitory activity of substituted aminooxadiazoles based on the proposed descriptors. The predictive ability of the transparent model MLR was excellent enough to be used to virtually screen novel PIM1 inhibitors from PubChem database.

Finally, we combined a machine learning approach using unambiguous MLR-QSAR model with PubChem database filtering concept to provide a rustic ligand-based virtual screening protocol. As a result, 16 potentially aminooxadiazole analogues as PIM1 inhibitors were identified. This study provides the theoretical basis and specific chemicals for PIM1 inhibitors, which can help the experimental research groups to search for potential anticancer.

## Abbreviations

QSAR: quantitative structure activity relationship
PIM: proviral integration site for Moloney murine leukaemia virus kinases
MLR: multiple linear regression
MNLR: multiple nonlinear regression
AD: applicability domain
GFA: Genetic Function Algorithm
Q^2^: cross-validated determination coefficient
R^2^: non-cross-validated correlation coefficient
MSE: standard error of the estimate
F: F test value
$R_{test}^{2}$: external validation determination coefficient
